# Channelization and flow depletion shift benthic macroinvertebrate and fish communities in urban rivers

**DOI:** 10.1371/journal.pone.0328843

**Published:** 2025-07-31

**Authors:** Shufeng Chen, Changcheng Guo, Xu Wang, Yalin Wu, Yidong Wang, Yinhua Wang, Hongyu Guo

**Affiliations:** 1 Beijing Municipal Research Institute of Eco-Environmental Protection, Beijing, China; 2 Tianjin Key Laboratory of Water Resources and Environment, Tianjin Normal University, Tianjin, China; 3 Tianjin Key Laboratory of Animal and Plant Resistance, College of Life Sciences, Tianjin Normal University, Tianjin, China; University of Mpumalanga, SOUTH AFRICA

## Abstract

Aquatic ecosystems worldwide are increasingly affected by human activities, with urbanization representing a major source of environmental stress. Channelization and flow depletion are key stressors in urban aquatic ecosystems. However, the combined effects of these factors on benthic macroinvertebrate and fish communities in urban rivers remain poorly understood. We examined the ecological impacts of channelization and flow depletion on benthic macroinvertebrates and fish in four urban rivers in Beijing, China: the natural high-flow Yongding River, the natural low-flow Gaojinggou River, the artificial high-flow Yongding River Diversion Channel, and the artificial low-flow Renmin Channel. By analyzing community composition, diversity, biomass, and water quality parameters, we assessed how river type (natural vs. artificial) and flow conditions (high vs. low) shape macroinvertebrate and fish communities across these urban rivers. Results showed that artificial channels had higher water temperatures, lower pH and DO, and higher concentrations of COD, NH_4_^+^, TP, fluorides, and sulfides compared to natural rivers, with flow depletion intensifying these effects. Both macroinvertebrate and fish community compositions varied significantly between river types and flow conditions. Channelization and flow depletion significantly reduced species richness, Shannon-Wiener diversity, and biomass in both macroinvertebrates and fish. Furthermore, we found a significant interaction between river type and flow depletion, as revealed by two-way ANOVA, with macroinvertebrate and fish communities in natural rivers being more sensitive to flow reductions than artificial channels. Redundancy analyses (RDAs) revealed that total phosphorus (TP) was the primary driver of macroinvertebrate community variation (contributing 23.6%), while DO played a crucial role in fish assemblages (contributing 20.6%). These findings underscore the significant impacts of channelization and flow depletion on urban river ecosystems, highlighting the vulnerability of natural rivers to flow depletion. Our study calls for urgent implementation of integrated management strategies to mitigate hydrological alterations, restore natural flow regimes, and reduce nutrient inputs, thereby enhancing the ecological resilience of urban aquatic ecosystems.

## Introduction

Globally, aquatic ecosystems are increasingly impacted by human activities [1]. Urbanization, as a significant form of human influence, reshapes aquatic ecosystems’ structure and function through multidimensional stresses [2–4]. Land use and cover changes directly contribute to river corridor fragmentation [5], with the expansion of impermeable surfaces exacerbating non-point source pollution [6]. Meanwhile, groundwater level declines and river diversion projects lead to reduced hydrological connectivity and water flow [7,8]. In this context, channelization and flow depletion have emerged as the most typical forms of disturbance to aquatic ecosystems caused by urbanization [9,10].

Natural rivers and artificial channels differ fundamentally in their hydro-morphological characteristics and ecological functions [11]. Natural rivers feature heterogeneous riverbed structures [12], dynamic hydrological conditions [13], and intact riparian vegetation zones [14], providing diverse microhabitats and niche differentiation for aquatic organisms [15,16]. In contrast, artificial channels, shaped by channelization, often have uniform geometries, homogeneous bed substrates, and hydrological rhythms regulated by weirs and dams [17], which may negatively affect aquatic communities [18]. River flow is a critical variable in aquatic ecosystems, with changes influencing a range of environmental factors that, in turn, affect the survival and growth of aquatic organisms [19]. In megacities, where water resources are becoming increasingly scarce, the significant reduction in river flow poses serious challenges to aquatic ecosystems [20].

Macroinvertebrates and fish are key functional groups within aquatic ecosystems, shaping river ecological patterns through material cycling, energy transfer, and food web regulation [21,22]. Macroinvertebrates, with their low mobility, serve as sensitive indicators of environmental changes, and their community composition reflects sediment quality and habitat stability [23,24]. Fish, as top consumers, regulate ecosystem structure and function through trophic cascades [25]. Together, these groups of aquatic organisms form the core biological indicators for assessing river ecological integrity [26]. However, the combined effects of channelization and flow depletion on macroinvertebrate and fish communities in urban river ecosystems remain unclear.

This study focused on four urban rivers in Beijing, China, representing different channelization and flow depletion conditions: natural high-flow (NH), natural low-flow (NL), artificial high-flow (AH), and artificial low-flow (AL). We analyzed the differences in benthic macroinvertebrate and fish community composition, diversity and growth among these urban rivers, investigating the combined effects of channelization and flow depletion on the aquatic communities. We hypothesized that channelization and flow depletion would significantly simplify benthic macroinvertebrate and fish community composition, leading to a decline in biodiversity and growth in these urban river ecosystems.

## Materials and methods

### Study sites

This study was conducted in four urban rivers under different human-induced channelization and water flow conditions: Yongding River (natural river with high-flow, NH), Gaojinggou River (natural river with low-flow, NL), Yongding River Diversion Channel (artificial channel with high-flow, AH), and Renmin Channel (artificial channel with low-flow, AL) in Shijingshan District, Beijing, China (S1 Table). The region was characterized by a temperate monsoon climate with a mean annual temperature of 12°C and precipitation of 640 mm yr^−1^ (approximately 70% in summer). The frost-free period and sunshine duration are approximately 200 days and 2600–2800 h yr^−1^, respectively.

The Yongding River, known as the mother river of Beijing, originates from the northern foothills of Guancen Mountain on the Shanxi Plateau. It stretches 747 km in length and drains a watershed area of 47,000 km². Within Shijingshan District, the river spans 12 km in length, with an average width of approximately 150 m. In July 2024, the Yongding River in Shijingshan District, Beijing, had a water flow of approximately 60 m³/s. The Gaojinggou River, a tributary of the Yongding River, stretches 2.5 km in length, with an average width of approximately 23 m and a mean water depth of about 1 m. In July 2024, the water flow of the Gaojinggou River in Shijingshan District, Beijing, was approximately 5 m³/s. The Yongding River Diversion Channel, the first water supply diversion project in Beijing, spans 25.38 km in length with an average width of approximately 150 m. In July 2024, the water flow in the channel within Shijingshan District was approximately 50 m³/s. The Renmin Channel, constructed for drainage purposes, stretches 4.76 km in length and has an average width of approximately 24 m. In July 2024, the water flow within the channel in Shijingshan District was approximately 5 m³/s. In each river, three representative sites were located for sampling.

### Water quality factor measurements

At each site, water samples were collected from five different locations. Water temperature was measured in situ using a thermometer (HT-YQ-273, Xinwang Instrument, China‌). Water pH was determined in situ using a portable pH meter (Star A 420C-01A, Thermo Orion, USA). Dissolved oxygen (DO) was measured in situ using a portable sensor (JPB-607A, Shanghai Leici Instrument, China). Water samples were brought back to the laboratory for the measurement of additional water quality parameters. Chemical oxygen demand (COD) was determined using titration with potassium dichromate (K_2_Cr_2_O_7_). Ammonium (NH_4_^+^) concentrations were measured using Nessler’s reagent spectrophotometry. Total phosphorus (TP) concentrations were measured using ammonium molybdate spectrophotometry. Sulfide concentrations were measured using methylene blue spectrophotometry. Spectrophotometric measurements were conducted using a UV spectrophotometer (UV-1800, Labsphere Instrument, China). Fluoride concentration was determined by a fluoride ion meter (MP519, Apera Instrument, China).

### Macroinvertebrate and fish community surveys

The investigation of macroinvertebrate and fish communities was conducted in July 2024. Three representative sampling sites were selected in each river, and sediment samples were collected from five different locations (including various microhabitats such as riffles, pools, and vegetation beds) at each site using a Petersen grab (0.0625 m²). The samples from these five locations were then combined to form a single composite sample for each site. The macroinvertebrate samples were obtained by sieving the sediment through a 0.5 mm mesh, and then preserved in a 10% formalin solution. For the fish community survey at each site, five sets of gillnets (each 20 m long and 2 m deep), each equipped with panels of different mesh sizes (2, 4, 6, 8, 10, 12, 15, and 18 cm between opposite knots), were deployed simultaneously at five different locations (including various microhabitats such as riffles, pools, and vegetation beds) from 18:00–08:00 the following day. The fish caught in the five sets were combined into a single composite sample for each site and preserved in a 10% formalin solution. Both macroinvertebrate and fish samples were brought back to the laboratory for classification, identification (genus or species level), and counting. The samples were then dried at 70°C for approximately 72 hours until a constant weight was achieved, and subsequently weighed to determine their dry biomass.

### Data analysis

We conducted ANOVAs after checking the data for normality and homogeneity of variance, followed by post hoc Tukey HSD tests (at a significance level of 0.05), using SPSS version 21. These analyses aimed to examine the effects of river type, river flow, and their interaction on water quality factors, as well as on the species richness, the Shannon-Wiener index, and the biomass of macroinvertebrates and fish. To compare community composition across different river types and flows, we performed non-metric multidimensional scaling (NMDS) analysis using PAST 4.09. Additionally, redundancy analysis (RDA) was performed to assess the relationship between the composition of macroinvertebrates, fish communities, and water quality factors using Canoco 5.0.

## Results

### Water quality factors across varied river types and flow conditions

As shown in Table 1, significant differences in water temperature were observed among different river types, with artificial channels leading to a marked increase in water temperature (S2 Table). Compared to natural rivers, artificial channels significantly reduced the pH of the water, and under lower flow conditions, the pH was notably lower than under higher flow conditions (S2 Table). DO levels in natural rivers were significantly higher than those in artificial channels, and under higher flow conditions, DO levels were significantly higher than under lower flow conditions (S2 Table). In artificial channels, concentrations of COD, NH_4_^+^, TP, fluoride, and sulfide were significantly higher than those in natural rivers, and under lower flow conditions, these factors were markedly higher than under higher flow conditions (S2 Table).

**Table 1:** Water quality factors across river types and flow conditions.

Water quality factors	NH	NL	AH	AL
Temperature (°C)	27.70 ± 0.75	28.37 ± 0.78	29.37 ± 0.38	31.10 ± 0.84
pH	8.60 ± 0.01	8.50 ± 0.01	8.23 ± 0.09	7.76 ± 0.09
DO (mg/L)	8.83 ± 0.13	7.54 ± 0.04	8.41 ± 0.09	7.27 ± 0.03
COD (mg/L)	11.67 ± 1.20	15.33 ± 1.20	18.00 ± 1.73	25.67 ± 1.45
NH_4_^+^ (mg/L)	0.13 ± 0.01	0.26 ± 0.02	0.55 ± 0.06	0.85 ± 0.05
TP (mg/L)	0.03 ± 0.01	0.06 ± 0.01	0.09 ± 0.01	0.13 ± 0.01
Fluoride (mg/L)	0.14 ± 0.02	0.24 ± 0.02	0.32 ± 0.05	0.42 ± 0.04
Sulfide (mg/L)	0.01 ± 0.01	0.02 ± 0.01	0.04 ± 0.01	0.06 ± 0.01

Note: NH, natural high-flow (Yongding River); NL, natural low-flow (Gaojinggou River); AH, artificial high-flow (Yongding River Diversion Channel); AL, artificial low-flow (Renmin Channel). DO, Dissolved Oxygen; COD, Chemical Oxygen Demand; TP, Total Phosphorus. The same below.

### Community compositions of macroinvertebrates and fish

**Fig 1:**
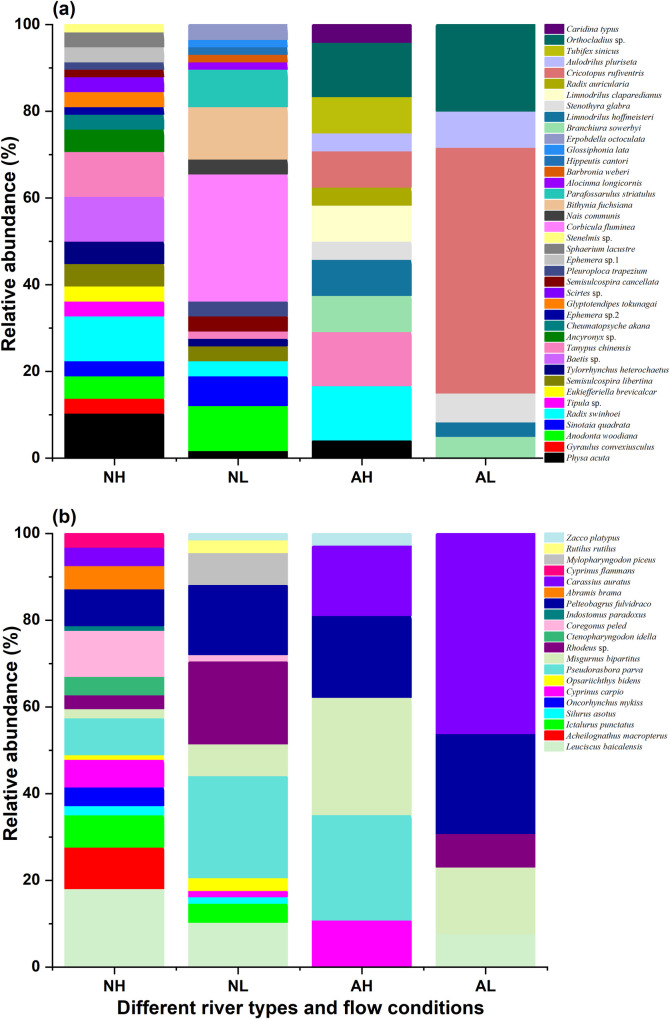
Community compositions of macroinvertebrates (a) and fish (b) across river types and flow conditions. NH, natural high-flow (Yongding River); NL, natural low-flow (Gaojinggou River); AH, artificial high-flow (Yongding River Diversion Channel); AL, artificial low-flow (Renmin Channel), The same below.

As shown in Fig 1a, a total of 21 macroinvertebrate species were found in the Yongding River (NH), with the highest proportions belonging to *Physa acuta*, *Radix swinhoei*, *Baetis* sp., and *Tanypus chinensis*, which are dominant species. In the Gaojing River (NL), 18 macroinvertebrate species were found, with *Corbicula fluminea* being the dominant species. In the Yongding River Diversion Channel (AH), 13 species were found, with *Orthocladius* sp., *Tanypus chinensis*, and *Radix swinhoei* being the dominant species. In the Renmin Channel (AL), 6 species were observed, with *Cricotopus rufiventris* being the dominant species.

Fig 1b showed that a total of 17 fish species were recorded in the Yongding River (NH), with *Hemiculter leucisculus* being the dominant species. In the Gaojing River (NL), 13 fish species were found, with *Pseudorasbora parva* as the dominant species. In the Yongding River Diversion Channel (AH) 6 species were observed, with *Misgurnus bipartitus* as the dominant species. In the Renmin Channel (AL), 5 species were recorded, with *Carassius auratus* being the dominant species.

**Fig 2:**
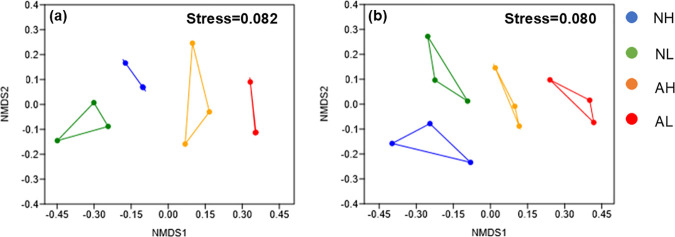
NMDS analyses of macroinvertebrate (a) and fish (b) community composition across river types and flow conditions (based on Bray-Curtis dissimilarity coefficient).

The results of the NMDS analysis are shown in Fig 2. It can be observed that the macroinvertebrate and fish communities exhibit clustering patterns according to different river types and flow conditions (NH, NL, AH and AL). The Stress values for the NMDS of macroinvertebrate and fish communities were 0.082 and 0.080, respectively, both below 0.1. This indicated that there were significant differences in the community composition of macroinvertebrates and fish across different river types and flow conditions.

### Relationship between macroinvertebrates, fish community composition, and water quality factors

**Fig 3:**
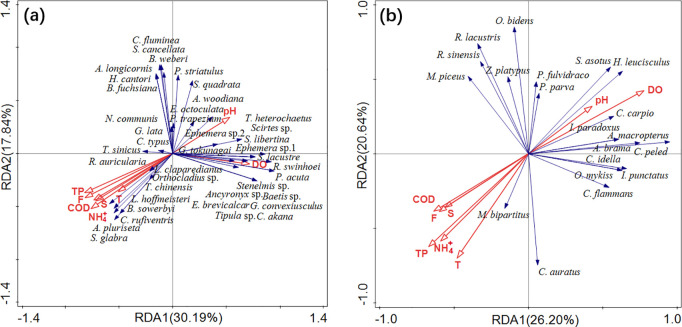
Redundancy analyses (RDAs) of benthic invertebrate (a) and fish (b) community compositions in relation to water quality factors across river types and flow conditions. T, Temperature; DO, Dissolved Oxygen; COD, Chemical Oxygen Demand; TP, Total Phosphorus; F, Fluoride; S, Sulfide.

Fig 3a showed that the eigenvalues of RDA1 and RDA2 are 30.19% and 17.84%, respectively, accounting for a total of 48.03% of the variance in the composition of benthic invertebrates. As shown in Table 2, total phosphorus concentration in the water (contributing 23.6% to the variation) is a key factor influencing the composition of benthic invertebrates (*P* < 0.05). The species *Radix auricularia*, *Tubifex sinicus*, *Caridina typus*, *Orthocladius* sp., *Limnodrilus hoffmeisteri*, *Branchiura sowerbyi*, *Cricotopus rufiventris*, *Stenothyra glabra*, and *Aulodrilus pluriseta* are positively correlated with total phosphorus concentration in the water.

**Table 2:** Contribution of water quality factors to the variation in macroinvertebrate and fish community composition in the RDAs.

Water quality factors	Macroinvertebrate		Fish	
	Contribution percentage (%)	*P*	Contribution percentage (%)	*P*
Temperature	9.3	0.342	6.5	0.490
pH	6.5	0.550	4.1	0.716
DO	9.1	0.232	20.6	0.002**
COD	6.6	0.524	6.2	0.550
NH_4_^+^	8.1	0.366	10.3	0.236
TP	23.6	0.002**	5.6	0.670
Fluoride	6.0	0.628	8.0	0.398
Sulfide	9.1	0.254	11.4	0.142

Note: *, 0.01 < *P* < 0.05; **, *P* < 0.01.

As shown in Fig 3b, the eigenvalues of RDA1 and RDA2 are 26.20% and 20.64%, respectively, accounting for a total of 46.84% of the variance in the composition of fish species. As shown in Table 2, dissolved oxygen concentration in the water (contributing 20.6% to the variation) is a key factor influencing the composition of fish species (*P* < 0.05). The species *Pseudorasbora parva*, *Silurus asotus*, *Hemiculter leucisculus*, *Cyprinus carpio*, *Indostomus paradoxus*, *Acheilognathus macropterus*, *Abramis brama*, *Coregonus peled*, *Ctenopharyngodon idella*, and *Ictalurus punctatus* are positively correlated with dissolved oxygen concentration in the water.

### Community diversity and biomass of macroinvertebrates and fish

**Fig 4:**
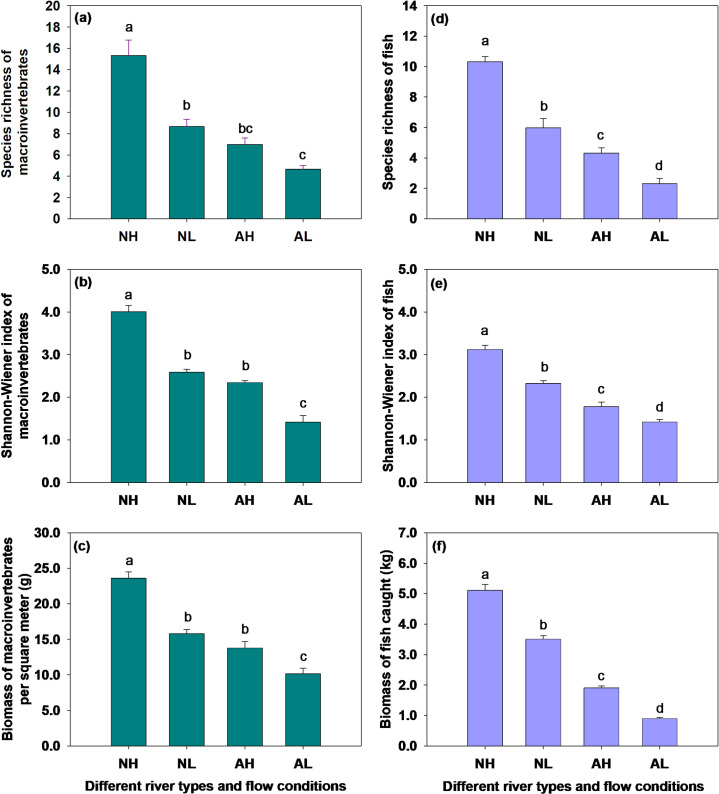
Species richness, Shannon-Wiener index and biomass of macroinvertebrates (a, b, c) and fish (d, e, f) across river types and flow conditions. Data are presented as means+SE (n = 3). Within each panel, shared letters indicate means that are not significantly different from each other (Tukey HSD tests, *P* < 0.05).

Compared to natural rivers, artificial channels significantly reduced the species richness of macroinvertebrates and fish. Similarly, lower river flows significantly decreased the species richness of both macroinvertebrates and fish, compared to higher river flows. Additionally, there was a significant interaction between river type and river flow. Flow depletion had a stronger negative effect on the species richness of macroinvertebrates and fish in natural rivers than in artificial channels (Fig 4a, Fig 4d and Table 3).

**Table 3:** Summary of ANOVAs examining the effects of river type, river flow and their interaction on species richness, Shannon-Wiener index, biomass of macroinvertebrate and fish communities.

Source of variance	df	*F*	*P*
Species richness of macroinvertebrate			
River type	1, 8	50.704	<0.001***
River flow	1, 8	27.000	<0.001***
River type × River flow	1, 8	6.260	0.037*
Shannon-Wiener index of macroinvertebrate			
River type	1, 8	162.341	<0.001***
River flow	1, 8	99.888	<0.001***
River type × River flow	1, 8	6.002	0.039*
Biomass of macroinvertebrate			
River type	1, 8	107.115	<0.001***
River flow	1, 8	58.575	<0.001***
River type × River flow	1, 8	7.871	0.023*
Species richness of fish			
River type	1, 8	140.167	<0.001***
River flow	1, 8	60.167	<0.001***
River type × River flow	1, 8	8.167	0.021*
Shannon-Wiener index of fish			
River type	1, 8	206.539	<0.001***
River flow	1, 8	53.998	<0.001***
River type × River flow	1, 8	7.789	0.024*
Biomass of fish			
River type	1, 8	734.538	<0.001***
River flow	1, 8	148.232	<0.001***
River type × River flow	1, 8	7.921	0.023*

Note: *, 0.01 < *P* < 0.05; **, *P* < 0.01; ***, *P* < 0.001.

In comparison to natural rivers, artificial channels significantly reduced the Shannon-Wiener diversity index of both macroinvertebrate and fish communities. Likewise, lower river flows significantly decreased the Shannon-Wiener diversity index of both macroinvertebrate and fish communities compared to higher flows. The interaction between river type and river flow was also significant, with flow depletion having a stronger negative impact on the Shannon-Wiener diversity index of both macroinvertebrate and fish communities in natural rivers compared to artificial ones (Fig 4b e and Table 3).

In contrast to natural rivers, artificial channels significantly reduced the biomass of both macroinvertebrate and fish communities. Lower river flows also significantly reduced the biomass of both macroinvertebrates and fish communities compared to higher flows. Furthermore, the interaction between river type and river flow was significant, with flow depletion having a stronger effect on the biomass of both macroinvertebrates and fish communities in natural rivers than in artificial ones (Fig 4c f and Table 3).

### Effects of water quality factors on diversity and biomass of macroinvertebrate and fish communities

**Fig 5:**
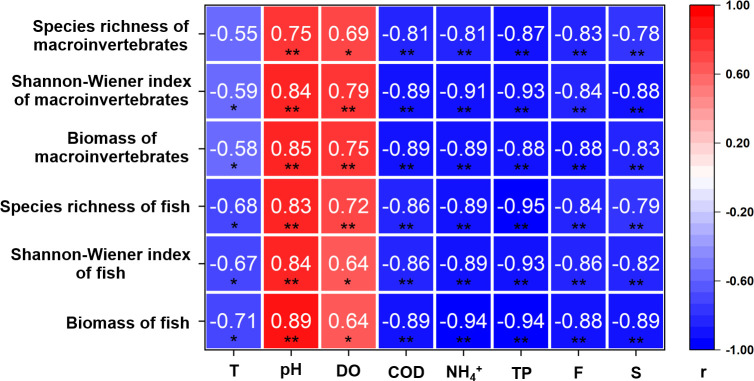
Correlations of species richness, Shannon-Wiener index, and biomass of macroinvertebrates and fish versus water quality factors across river types and flow conditions. Water quality factors: T (Temperature), pH, DO (Dissolved Oxygen), COD (Chemical Oxygen Demand), NH_4_^+^, TP (Total Phosphorus), F (Fluoride), S (Sulfide). Pearson’s r values are shown; *, 0.01 < *P* < 0.05; **, *P* < 0.01.

Fig 5 showed that an increase in water temperature had a significant negative impact on the Shannon-Wiener diversity index and biomass of macroinvertebrate communities (*P* < 0.05). It also negatively affected the species richness, Shannon-Wiener diversity index, and biomass of fish communities (*P* < 0.05). In contrast, increases in water pH and dissolved oxygen (DO) levels had positive effects on the species richness, Shannon-Wiener diversity index, and biomass of both macroinvertebrate and fish communities (*P* < 0.05). On the other hand, increases in chemical oxygen demand (COD), NH_4_^+^, total phosphorus (TP), fluoride, and sulfide concentrations all had a significant negative effect on the species richness, Shannon-Wiener diversity index, and biomass of both macroinvertebrate and fish communities (*P* < 0.01).

## Discussion

### Effects of channelization on the community composition, diversity, and growth of macroinvertebrates

The results of this study showed that river channelization in an urban area significantly altered macroinvertebrate communities, reducing both biodiversity and biomass compared to natural river systems. This is probably due to the structural interventions typically involved in river channelization, such as widening, deepening the riverbed, and constructing embankments [18]. These modifications considerably change the river’s hydrodynamic characteristics and overall ecological environment [27].

First, channelization can lead to changes in flow velocity and flow patterns. In natural rivers, flow velocity and direction vary greatly, whereas in artificially modified channels, the flow tends to be more uniform and faster [28]. This change may affect the habitat selection of macroinvertebrates, particularly those relying on still or slow-moving waters [29]. These species may face habitat loss or struggle to adapt to the modified environment, leading to shifts in community composition and reduction in both diversity and biomass.

Moreover, channelization often results in changes to the riverbed substrate. Artificial modifications typically cause a redistribution or accumulation of sediments such as sand, gravel, and clay [30], which can affect the habitat and feeding grounds for benthic macroinvertebrates [31]. Many of these organisms depend on the type and structure of the riverbed to provide suitable living spaces and food sources [32]. Therefore, changes in the riverbed can create selective pressures on certain species, making them unable to adapt to the new conditions, thereby altering the community structure and reducing diversity and biomass.

Additionally, channelization can impact water quality, further affecting the health of the river ecosystem. As shown in this study, channelized rivers may undergo water quality degradation, such as increased pollutant loads and reduced DO levels (Table 1). This would negatively impact the survival and growth of some macroinvertebrate species [33], thereby altering macroinvertebrate community composition and reducing both diversity and biomass of macroinvertebrates. At the same time, degradation of water quality induced by channelization would also affect the supply of organic matter [34], which serves as a key food source for macroinvertebrates. The reduction in organic matter can lead to increased competition for resources, potentially limiting the reproduction or growth of certain macroinvertebrate species. This may lead to shifts in community composition and reductions in both diversity and biomass.

Specifically, we found that the concentrations of total phosphorus (TP) in the rivers significantly affected the community composition of benthic macroinvertebrates. As a key indicator of nutrient enrichment, TP could lead to shifts in macroinvertebrate community structure by promoting the growth of algae and aquatic plants [35], which might in turn alter the availability of resources and habitats for macroinvertebrates. Our findings suggested that managing TP levels is crucial to maintaining healthy benthic macroinvertebrate populations and preserving aquatic ecosystem integrity in urban rivers.

### Effects of channelization on the community composition, diversity, and biomass of fish

This study found that channelization significantly altered the fish community compositions and reduced fish diversity and biomass in the urban rivers, which could be influenced by changes in hydrological patterns. In natural rivers, water flow is variable and diverse, providing different habitats for various fish species, such as riffles, pools, and slackwater areas [36]. In contrast, channelized rivers tend to have more uniform and straightened water flow, reducing hydrological habitat diversity [37]. This change may result in some fish species losing their hydrological habitat or being unable to adapt to the new environmental conditions [38], leading to significant shifts in community composition and reduction in diversity and biomass.

The changes in fish community composition and the decline in fish diversity and biomass in channelized rivers may also result from the ecological degradation induced by channelization. In natural rivers, complex hydrological environments and diverse aquatic vegetation offer abundant habitats and food resources for a wide range of fish species [39]. However, channelization usually leads to straightened riverbanks, altered bed substrates, increased flow velocities, and reduced vegetation cover, all of which decrease habitat complexity and thereby negatively affecting fish survival and biomass [40]. Consequently, many fish species may decline due to unsuitable habitats, leading to alteration in community compositions and decreases in diversity and biomass.

The shifts in fish community composition, along with declines in diversity and biomass, can also be attributed to the impact of channelization on water quality. The results of this study showed that channelization could change water quality (Table 1), and previous research has indicated that water quality deterioration would negatively impact the survival and growth of fish species [41]. Consequently, the water quality degradation caused by channelization could contribute to the loss or stunted growth of certain fish species, leading to shifts in community composition and declines in both diversity and biomass. In particular, we found that dissolved oxygen (DO) levels significantly influenced the composition of fish communities. As DO is essential for fish respiration, a reduction in its availability can induce physiological stress, leading to shifts in fish species distributions [42]. Species with higher oxygen demands may be displaced by more tolerant species, altering community structure [43]. These findings underscore the importance of maintaining sufficient oxygen levels in aquatic ecosystems to support healthy fish communities and preserve the ecological integrity of urban rivers.

Moreover, as shown in this study (Fig 5), the degradation of water quality caused by channelization would negatively affect the communities of benthic organisms. While, these organisms are important food sources for fish [44], so their decreased abundance would directly negatively affect fish survival and growth, resulting in reduction in diversity and biomass.

Additionally, channelization can disrupt ecological connectivity, which can obstruct fish migration routes, particularly for species with strong migratory tendencies. This prevents fish from accessing suitable spawning grounds, ultimately altering community composition and reducing diversity and biomass [45]. In natural river systems, ecological connectivity provides pathways for species migration and genetic exchange [46]. However, channelization often breaks this connectivity, limiting fish species’ ability to expand their populations, which reduces genetic diversity and, consequently, the species’ survival potential [47]. Thus, loss of connectivity induced by channelization may be an important factor in the reduction of fish diversity and biomass, as it limits species’ access to survival spaces and food resources.

### Effects of waterflow on the community composition, diversity, and biomass of macroinvertebrates

In this study, we found that water flow changes significantly altered the community composition of benthic macroinvertebrates, and that reduction in water flow significantly decreased the diversity and biomass of these organisms. Several ecological mechanisms may underlie these results. First, changes in water flow would directly affect the habitat of benthic invertebrates. Natural river flows are typically complex and diverse, providing a variety of habitats, such as fast-flowing areas and calm pools, where different macroinvertebrate species can find ecological niches that support their survival and growth [48]. However, reduced water flow may result in habitat homogenization, diminishing habitat complexity [49] and, as a result, altering the macroinvertebrate community composition while decreasing both diversity and biomass of macroinvertebrates.

Moreover, a decrease in water flow may impact the food supply for benthic macroinvertebrates. Water flow helps distribute nutrients and organic materials, increasing the availability of phytoplankton and organic detritus [50], which are important food sources for benthic macroinvertebrates. A reduction in water flow could limit food resources for benthic macroinvertebrates, thereby impacting their survival and growth [51], ultimately leading to shifts in community composition and a decline in both diversity and biomass.

In addition, another underlying mechanism behind these findings may be the degradation of water quality associated with reduced water flow (Table 1). Lower water flow can lead to the stagnation of water in certain river sections, which can negatively impact the distribution and dilution of nutrients and organic matter [52]. In fast-flowing rivers, water movement helps to maintain a balance by dispersing nutrients, oxygen, and organic materials, thereby preventing the accumulation of pollutants and organic matter [53]. However, in low-flow conditions, the reduced water movement can result in the concentration of nutrients such as nitrogen and phosphorus, leading to eutrophication [54]. This process can cause the growth of harmful algae and oxygen depletion, which limits the survival and growth of macroinvertebrates [55]. As a result, it leads to shifts in macroinvertebrate community composition and a reduction in both their diversity and biomass.

### Effects of waterflow on the community composition, diversity, and biomass of fish

In this study, we found that reduced water flow significantly altered fish community composition, and led to a substantial decline in both fish diversity and biomass. These findings could be attributed to the following mechanisms. First, reduced water flow directly affects river habitat structure. In rivers with high flow, the varied hydrological conditions, such as deep pools, rapids, and shallows, maintain habitat diversity, providing different types of environments for fish species [56]. A decrease in flow may lead to the loss or alteration of these habitats, making it difficult for fish species with specific flow requirements to survive [57]. Therefore, habitat degradation is a key mechanism by which reduced water flow affects fish community structure, resulting in decreased diversity and biomass.

Moreover, a decrease in water flow may also lead to the deterioration of water quality, thereby negatively impacting fish survival and biomass. In low-flow conditions, concentrations of nutrients, pollutants, and organic materials in the water would increase (Table 1), which likely leads to eutrophication [58]. This could cause algal blooms, further reducing oxygen levels in the water, especially in slower-flowing areas where oxygen exchange is less efficient [59]. Low-oxygen environments pose a significant threat to fish, particularly species that require high oxygen levels [60]. The deterioration of water quality not only affects fish survival but also alters fish habitat preferences and the food chain structure [61], further shifting the composition of fish communities, and reducing fish diversity and biomass.

In addition, reduced flow can also influence competition and predation pressures among fish species [62]. In faster-flowing environments, food distribution is generally more even, and predation is relatively stable [63]. However, under low-flow conditions, food resources may become concentrated in certain areas or scarce, increasing competition among species [64]. Some fish species may decline due to poor competition ability, while more competitive species may thrive, leading to shifts in community composition and declines in both fish diversity and biomass.

### Interaction between channelization and flow depletion on macroinvertebrate and fish communities

This study revealed significant interaction between channelization and flow depletion on macroinvertebrate and fish community characteristics (Table 3), indicating notable differences in how these communities respond to flow depletion in natural versus artificial rivers. Natural rivers usually have more ecological complexity, with more varied hydrological conditions, including fast currents, slower-moving sections, deep zones, and shallow areas [65]. These diverse habitats may provide suitable environments for various aquatic organisms [66,67]. When water flow is reduced, the structure and distribution of these habitats are significantly affected, leading to a decline in biodiversity and growth of aquatic species. In contrast, artificial rivers (such as channelized rivers) often lack this complex hydrological structure and have been altered by human intervention [68], typically featuring a more uniform flow with higher speed. Therefore, the impact of water flow reduction in artificial rivers may be relatively weaker, primarily resulting in the deterioration of specific habitat types, such as aquatic vegetation or riverbed communities, rather than a complete loss of ecological complexity.

Moreover, in natural rivers, a reduction in water flow may not only affect aquatic habitats but also directly disrupt ecological processes driven by the water flow, such as nutrient cycling, material transport, and species interactions [69]. Flow depletion can lead to habitat shrinkage, particularly for aquatic species dependent on high flow speeds or specific hydrological conditions for feeding and reproduction, resulting in a significant decline in both diversity and growth [70]. In comparison, artificial rivers have more uniform hydrological conditions, and many ecological processes have already been simplified or lost due to human intervention [27]. As a result, ecosystems in artificial rivers tend to be more resilient to water flow reductions, although biodiversity loss and biomass declines of aquatic species still occur.

In addition, natural rivers can support greater species diversity and complex biological interactions [71,72], so reduced water flow often disrupts the ecological balance, altering competitive relationships between species [73,74]. Species unable to adapt may decline, while more competitive species may dominate, leading to shifts in community composition and reduced diversity. In contrast, in channelized rivers, where species composition has already been strongly negatively affected by human activities and hydrological conditions are simplified [75], the impact of flow reduction on community structure and diversity of aquatic species would be generally weaker.

## Conclusion

In conclusion, our study revealed the significant ecological consequences of channelization and flow depletion on macroinvertebrate and fish communities in urban rivers. The results demonstrated that both channelization and flow depletion could significantly shift macroinvertebrate and fish community composition, reducing both the diversity and biomass of these aquatic organisms. Additionally, we found that there was a significant interaction between river type and flow depletion. Macroinvertebrate and fish communities in natural rivers were more sensitive to flow reductions than those in channelized rivers. These findings emphasize the urgent need for integrated management strategies, such as those combining habitat restoration, water quality improvement, and flow regulation, to address both river modifications and hydrological changes. This approach is essential to preserve biodiversity and ecosystem function in urban river ecosystems, with particular focus on the ecological conservation of natural rivers in urban areas.

## Supporting information

S1 Table Geographical coordinates of the study sites in the four urban rivers. The rivers are Yongding River, Gaojinggou River, Yongding River Diversion Channel, and Renmin Channel in Shijingshan District, Beijing, China.
(DOCX)

S2 Table Summary of ANOVAs examining the effects of river type, river flow and their interaction on water quality factors. (DOCX)
