# Supplementary material for: Channelization and flow depletion shift benthic macroinvertebrate and fish communities in urban rivers
Source: PLoS One. 2025 Jul 31;20(7):e0328843. doi: 10.1371/journal.pone.0328843 (PMC12312919; doi:10.1371/journal.pone.0328843)
Supplement: S1 Table — The rivers are Yongding River, Gaojinggou River, Yongding River Diversion Channel, and Renmin Channel in Shijingshan District, Beijing, China. (DOCX) [file pone.0328843.s001.docx]

**Supporting information**

**Channelization and flow depletion shift** **benthic macroinvertebrate and fish communities in urban rivers**

Shufeng Chen^1^, Changcheng Guo^2^, Xu Wang^1^, Yalin Wu^1^, Yidong Wang^2^,

Yinhua Wang^3,*^, Hongyu Guo^3,*^

1. Beijing Municipal Research Institute of Eco-Environmental Protection, Beijing 100037, China

2. Tianjin Key Laboratory of Water Resources and Environment, Tianjin Normal University, Tianjin 300387, China

3. Tianjin Key Laboratory of Animal and Plant Resistance, College of Life Sciences, Tianjin Normal University, Tianjin 300387, China

*Corresponding authors: Yinhua Wang, email: [wangyinhua@tjnu.edu.cn](mailto:wangyinhua@tjnu.edu.cn);

Hongyu Guo, email: [skyghy@tjnu.edu.cn](mailto:skyghy@tjnu.edu.cn)

**Table S1.** **Geographical coordinates of the study sites in the four urban rivers.** The rivers are Yongding River, Gaojinggou River, Yongding River Diversion Channel, and Renmin Channel in Shijingshan District, Beijing, China.

| **River** | **River type and river flow** | **Site** | **Longitude** | **Latitude** |
| --- | --- | --- | --- | --- |
| Yongding River | natural river with high-flow (NH) | YD1 | E 116.169101 | N 39.975851 |
|  |  | YD2 | E 116.166531 | N 39.975759 |
|  |  | YD3 | E 116.166530 | N 39.975259 |
|  |  |  |  |  |
| Gaojinggou River | natural river with low-flow (NL) | GJ1 | E 116.140533 | N 39.910018 |
|  |  | GJ2 | E 116.173499 | N 39.938083 |
|  |  | GJ3 | E 116.185311 | N 39.939593 |
|  |  |  |  |  |
| Yongding River Diversion Channel | artificial channel with high-flow (AH) | YY1 | E 116.168031 | N 39.975509 |
|  |  | YY2 | E 116.126562 | N 39.924709 |
|  |  | YY3 | E 116.131229 | N 39.921811 |
|  |  |  |  |  |
| Renmin Channel | artificial channel with low-flow (AL) | RM1 | E 116.194429 | N 39.939541 |
|  |  | RM2 | E 116.124548 | N 39.929789 |
|  |  | RM3 | E 116.124748 | N 39.929181 |
