# Supplementary material for: Channelization and flow depletion shift benthic macroinvertebrate and fish communities in urban rivers
Source: PLoS One. 2025 Jul 31;20(7):e0328843. doi: 10.1371/journal.pone.0328843 (PMC12312919; doi:10.1371/journal.pone.0328843)
Supplement: S2 Table — (DOCX) [file pone.0328843.s002.docx]

**Supporting information**

**Channelization and flow depletion shift** **benthic macroinvertebrate and fish communities in urban rivers**

Shufeng Chen^1^, Changcheng Guo^2^, Xu Wang^1^, Yalin Wu^1^, Yidong Wang^2^,

Yinhua Wang^3,*^, Hongyu Guo^3,*^

1. Beijing Municipal Research Institute of Eco-Environmental Protection, Beijing 100037, China

2. Tianjin Key Laboratory of Water Resources and Environment, Tianjin Normal University, Tianjin 300387, China

3. Tianjin Key Laboratory of Animal and Plant Resistance, College of Life Sciences, Tianjin Normal University, Tianjin 300387, China

*Corresponding authors: Yinhua Wang, email: [wangyinhua@tjnu.edu.cn](mailto:wangyinhua@tjnu.edu.cn);

Hongyu Guo, email: [skyghy@tjnu.edu.cn](mailto:skyghy@tjnu.edu.cn)

**Table S2. Summary of ANOVAs examining the effects of river type, river flow and their interaction on water quality factors.**

| **Source of variance** | **df** | ***F*** | ***P*** |
| --- | --- | --- | --- |
| Temperature |  |  |  |
| River type | 1, 8 | 6.764 | 0.032* |
| River flow | 1, 8 | 2.012 | 0.193 |
| River type × River flow | 1, 8 | 0.398 | 0.546 |
| pH |  |  |  |
| River type | 1, 8 | 77.786 | <0.001*** |
| River flow | 1, 8 | 20.643 | 0.002** |
| River type × River flow | 1, 8 | 8.643 | 0.019* |
| DO |  |  |  |
| River type | 1, 8 | 17.296 | 0.003** |
| River flow | 1, 8 | 206.465 | <0.001*** |
| River type × River flow | 1, 8 | 0.858 | 0.381 |
| COD |  |  |  |
| River type | 1, 8 | 52.083 | <0.001*** |
| River flow | 1, 8 | 24.083 | 0.001** |
| River type × River flow | 1, 8 | 3.000 | 0.122 |
| NH_4_^+^ |  |  |  |
| River type | 1, 8 | 137.440 | <0.001*** |
| River flow | 1, 8 | 23.804 | 0.001** |
| River type × River flow | 1, 8 | 3.925 | 0.083 |
| TP |  |  |  |
| River type | 1, 8 | 62.228 | <0.001*** |
| River flow | 1, 8 | 16.409 | 0.004** |
| River type × River flow | 1, 8 | 0.046 | 0.837 |
| Fluoride |  |  |  |
| River type | 1, 8 | 24.178 | 0.001** |
| River flow | 1, 8 | 7.634 | 0.025* |
| River type × River flow | 1, 8 | 0.002 | 0.964 |
| Sulfide |  |  |  |
| River type | 1, 8 | 36.000 | <0.001*** |
| River flow | 1, 8 | 11.111 | 0.010* |
| River type × River flow | 1, 8 | 1.778 | 0.219 |

Note: *, 0.01<*P*<0.05; **, *P*<0.01; ***, *P*<0.001
